# Atypical Electrocardiogram in Acute Pericarditis: A Case Report

**DOI:** 10.7759/cureus.42888

**Published:** 2023-08-03

**Authors:** Aymen Basha, Marina Raikhel, Syed Hussaini, Parinaz Afrashteh

**Affiliations:** 1 Family Medicine, Mission Community Hospital, Los Angeles, USA; 2 Family Medicine Private Practice Physician, Torrance Lomita Medical Center, Lomita, USA

**Keywords:** pericardial friction rub, atypical electrocardiogram, atypical pericarditis, pr-segment depression, st-segment elevation, acute pericarditis

## Abstract

Pericarditis is a disorder defined by inflammation of the pericardium, commonly presenting with chest pain, fever, and pericardial friction. Electrocardiography (EKG) is frequently utilized to diagnose pericarditis, as it frequently reveals particular changes like ST-segment elevations and PR-segment depressions. Nevertheless, there are cases where individuals show all symptoms of pericarditis yet present an atypical or irregular EKG. This case report intends to highlight the case of a patient who came to an outpatient medical facility with all the common symptoms of pericarditis yet presented an atypical electrocardiogram. Moreover, this report intends to look into the hypothesis that in patients showing symptoms of pericarditis but with an atypical EKG, we might be looking at an irregular or even non-specific variant of pericarditis. This highlights the value of an extensive diagnostic approach consisting of high-quality imaging studies, such as transthoracic echocardiography, as well as medical assessments if an EKG does not give definite proof of pericarditis.

## Introduction

Pericarditis is the inflammation of the pericardium, which is a sac-like membrane layer that covers the heart. The pericardium consists of two layers with fluid in between them to enable the heart to beat comfortably. Pericarditis could be broken down into acute pericarditis, subacute pericarditis, chronic pericarditis, and recurrent pericarditis. Patients with pericarditis usually complain of stabbing and sharp retrosternal or left-sided chest pain. The pain is generally aggravated by lying down and gets better by leaning forward. A friction rub, which is a pathognomonic scratching sound heard on auscultation of the heart, is commonly present in most patients with pericarditis [1]. 

Pericarditis can present itself in many types and forms. Fibrinous pericarditis results from fibrin layer formation on an inflamed pericardium, resulting in cardiac dysfunction. It commonly occurs as a result of trauma, surgery, acute myocardial infarction, uremia, collagen vascular disorders, and malignancies [2]. Purulent pericarditis is primarily the result of a bacterial infection. The spread of the infection into the pericardial space results in purulent fluid formation and inflammation of the pericardium. Treatment includes drainage of the pericardial space along with the administration of systemic antibiotics [3]. Constrictive pericarditis is a result of chronic inflammation, thickening, fibrosis, and calcification of the pericardium that leads to diastolic dysfunction of the heart [4]. Dressler syndrome is a form of pericarditis that often occurs as a complication of myocardial infarction, post-cardiac surgery, or as a result of a traumatic injury to the heart. It is believed to occur as a result of anti-myocardial antibodies that are formed due to a delayed autoimmune process [1].

Pericarditis can develop from a wide range of causes, of which infectious causes are known to be a very common etiology. Viruses are considered the most prevalent cause of pericarditis. Coxsackieviruses A and B, adenovirus, parvovirus B19, HIV, and influenza are notably among the most common causes of pericarditis. On the other hand, bacterial causes of pericarditis are known to occur less frequently, with *Mycobacterium tuberculosis* noted as one of the leading bacterial causes of pericarditis worldwide. Other less common causes of bacterial pericarditis include *Staphylococcus*, *Streptococcus*, *Pneumococcus*, and *Meningococcus*. Fungal causes of pericarditis are known to be extremely rare and primarily occur in immunocompromised individuals. The most notable organisms include *Histoplasma*, *Coccidiodes*, *Candida*, and *Blastomycosis* [1]. 

Non-infectious causes of pericarditis include connective tissue diseases such as systemic lupus erythematosus and rheumatoid arthritis, metabolic causes such as uremia and myxedema, and malignancy. Trauma is also known to be a common etiology of pericarditis. Other post-cardiac injuries known to lead to pericarditis include percutaneous intervention, cardiac surgery, or direct blunt trauma to the chest. Last, medications are also known to induce pericarditis. A wide range of medications have been found to cause drug-induced pericarditis, with hydralazine, procainamide, and isoniazid among the most notable medications [1]. 

Recognizing the wide range of etiologies of pericarditis is essential for proper diagnosis and treatment. A thorough investigation, including an extensive patient history, risk factors, previous diagnosis, treatments, surgeries, and medication list, can play a vital role in understanding and identifying the underlying etiology. Laboratory workup, imaging studies, and pericardial fluid analysis can aid in establishing a diagnosis of pericarditis. Although various etiologies of pericarditis are known, in up to 90% of cases, no clear cause can be established, in which case a diagnosis of idiopathic pericarditis is often made [1].

## Case presentation

A 67-year-old female visited her primary care physician with complaints of acute-onset chest pain aggravated by normal respirations and changes in body position. The chest pain is described as "sharp and stinging." Moreover, the pain is associated with shortness of breath, orthopnea, and fatigue. The patient mentioned that sleeping on two pillows helps with the symptoms. Physical examination showed mild edema of the lower extremities and a pericardial friction rub, consistent with acute pericarditis. There were no signs of carotid bruits or increased jugular venous pressure indicating internal carotid artery disease or right heart failure, respectively. 

**Figure 1:**
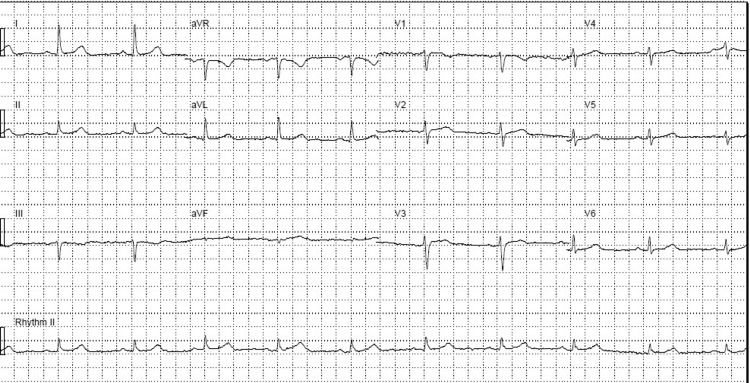
Initial EKG shows normal sinus rhythm without ST-segment elevations or PR-segment depressions

Despite the classic pericarditis symptoms, the EKG showed a normal sinus rhythm without any ST-segment elevations or PR-segment depressions (Figure 1). A full blood panel was ordered to check for inflammatory markers such as C-reactive protein (CRP).

As a result of the disparity between the patient's classic pericarditis symptoms and the atypical EKG findings, the patient was referred to a cardiologist for additional medical care. A transthoracic echocardiogram is ordered to check for pericardial effusion and the presence of pericardial inflammation. Cardiac MRI will also be utilized to check for indications of pericardial thickening and validate the medical diagnosis of acute pericarditis.

## Discussion

Contrasting this case with a research study that was done on 44 patients with acute pericarditis, our team confirmed that irregular EKG in acute pericarditis is common since it occurred in 43% of the study's participants [5]. To become included in their research study, patients had to meet particular requirements, including the presence of symptoms, stage one EKG abnormalities, and an unequivocal pericardial rub. Participants were excluded from the study in the absence of symptoms or pericardial rub. The EKGs were categorized as typical or typical variants based on the presence of generalized ST-segment elevations involving both limb and precordial lead tracings. Atypical EKGs were defined as those exhibiting any ST segment abnormalities other than typical deviations, different distributions of ST deviations, or a complete absence of ST segment deviations. The presence of a pericardial rub was a requirement for patients with atypical EKGs. The absence of EKG changes could be deceptive, highlighting the importance of a pericardial rub as a diagnostic factor [5].

The research study additionally reviewed PR-segment deviations, which were observed in 14 patients with typical EKGs and 14 patients with atypical EKGs. They found that in four cases with atypical EKGs, PR-segment changes were the only EKG abnormality found. These findings highlight the value of PR-segment changes, as they can provide valuable clues in the absence of other EKG changes [5].

Taking into account those research study results, it is important to take into consideration the possible underlying etiologies of pericarditis. While viral infections are the most common cause, other factors such as autoimmune diseases, recent heart surgery, and certain medications such as hydralazine and procainamide could also contribute [1]. Establishing the main etiology is important for creating a suitable treatment plan and management strategy tailored to the patient's condition.

As soon as the medical diagnosis of pericarditis is validated, the treatment and management approach will depend on the underlying etiology as well as the severity of the symptoms. Nonsteroidal anti-inflammatory drugs (NSAIDs) like ibuprofen or aspirin are usually prescribed as first-line therapy to relieve pain and reduce inflammation. Nevertheless, taking into consideration the patient's age and medical history, the use of NSAIDs would be evaluated, and alternative options such as corticosteroids might be considered if necessary. Close monitoring and regular follow-up will be required to assess the patient's response to treatment and ensure a favorable prognosis [1].

The prognosis for pericarditis varies based on different factors, including the underlying etiology and the response to treatment. While most cases of acute pericarditis resolve spontaneously or with medical management, complications such as pericardial effusion, cardiac tamponade, or chronic and recurrent pericarditis can occur. Thus, ongoing monitoring and appropriate intervention are essential to ensuring a positive outcome for the patient [1].

## Conclusions

In conclusion, this case report highlights pericarditis as the main diagnosis, although the patient had atypical EKG findings. Atypical variants of pericarditis could be a diagnostic challenge for physicians, especially when patients show all signs and symptoms of the disorder yet present an atypical or irregular EKG. This case report stresses the necessity for a comprehensive diagnostic approach, such as imaging studies and medical assessments, to precisely diagnose acute pericarditis in similar cases. Further research is required to better understand the underlying mechanisms and medical implications of atypical variants of pericarditis.
